# Beyond Penicillin: The Potential of Filamentous Fungi for Drug Discovery in the Age of Antibiotic Resistance

**DOI:** 10.3390/antibiotics12081250

**Published:** 2023-07-29

**Authors:** João Correia, Anabela Borges, Manuel Simões, Lúcia C. Simões

**Affiliations:** 1 LEPABE—Laboratory for Process Engineering, Environment, Biotechnology and Energy, Faculty of Engineering, Department of Chemical Engineering, University of Porto, 4200-465 Porto, Portugal; up201806894@edu.fe.up.pt (J.C.); apborges@fe.up.pt (A.B.); mvs@fe.up.pt (M.S.); 2 ALiCE—Associate Laboratory in Chemical Engineering, Faculty of Engineering, University of Porto, 4200-465 Porto, Portugal; 3 CEB—Centre of Biological Engineering, University of Minho, Campus de Gualtar, 4710-057 Braga, Portugal; 4 LABBELS—Associate Laboratory in Biotechnology, Bioengineering and Microelectromechanical Systems, 4710-057 Braga, Portugal

**Keywords:** antibiotic resistance, biofilm control, antibiotic bioprospection, bioprocess, filamentous fungi

## Abstract

Antibiotics are a staple in current medicine for the therapy of infectious diseases. However, their extensive use and misuse, combined with the high adaptability of bacteria, has dangerously increased the incidence of multi-drug-resistant (MDR) bacteria. This makes the treatment of infections challenging, especially when MDR bacteria form biofilms. The most recent antibiotics entering the market have very similar modes of action to the existing ones, so bacteria rapidly catch up to those as well. As such, it is very important to adopt effective measures to avoid the development of antibiotic resistance by pathogenic bacteria, but also to perform bioprospecting of new molecules from diverse sources to expand the arsenal of drugs that are available to fight these infectious bacteria. Filamentous fungi have a large and vastly unexplored secondary metabolome and are rich in bioactive molecules that can be potential novel antimicrobial drugs. Their production can be challenging, as the associated biosynthetic pathways may not be active under standard culture conditions. New techniques involving metabolic and genetic engineering can help boost antibiotic production. This study aims to review the bioprospection of fungi to produce new drugs to face the growing problem of MDR bacteria and biofilm-associated infections.

## 1. The Rise of Bacterial Resistance

During most of human history, infectious diseases such as smallpox, cholera, diphtheria, pneumonia, typhoid fever, plague, tuberculosis, typhus, and syphilis were the leading cause of mortality, even after the world industrialized. Antibiotics revolutionized the way infections were treated, saving millions of lives and shifting the leading causes of death to non-communicable diseases like cardiovascular disease, cancer, and stroke [1]. Antibiotics also allowed for the improvement of other procedures of great importance in modern medicine, lowering the risks of organ transplants, childbirth, and chemotherapy [2].

Even in the early days of readily available antibiotics, it was observed that bacteria could develop resistance to these drugs, which is a phenomenon that has become a significant public health concern in modern times. Sir Alexander Fleming warned that when antibiotics are used too often or in improper amounts, modifications in bacteria may be induced, rendering them resistant to these drugs [3]. However, from 1944 to the 1980s, the discovery of new molecules occurred at a rapid pace, which effectively rendered bacterial resistance less of a concern. This is because new resistant strains appeared at a much slower rate compared to the frequent discovery of new antibiotic molecules, leading to the “golden age” of antibiotics [4]. More recently, drug development has been much slower, and has been more focused on making alterations to previously known molecules instead of developing new modes of action. In circa 50 years, only two classes of antibiotics were discovered and advanced for clinical use—oxazolidinones and lipopeptides [5,6,7,8]. Bacteria thus had time to become resistant to antibiotics, sometimes to those belonging to more than one class. These multi-drug-resistant (MDR) bacteria have been of increasing concern, and in some cases, they may accumulate resistance to almost all available antibiotics, the so-called superbugs or pandrug-resistant bacteria [1]. Tigecycline, colistin, daptomycin, vancomycin, and linezolid are last-resource antibiotics due to their potent antimicrobial activity. However, currently, the alarming increase in antibiotic-resistant bacteria has rendered these drugs ineffective in a growing number of cases [9,10,11,12]. According to an influential study conducted in 2014, it was estimated that altogether, bacterial infections will kill 10 million people annually by 2050, surpassing the number of deaths that are currently caused by cancer [13]. The assumptions made to calculate this number are often overlooked. It is a prediction of what will happen if no immediate action takes place, but actions have been taken. However, even if this number of victims is no longer likely in this time frame, antibiotic resistance is a growing problem that requires an active, continuous effort to avoid a critical scenario in just a few decades [14].

The growing incidence of MDR bacteria has many causes. Antibiotics are found in the wild, so some bacteria are naturally resistant to them, even before the modern era of antibiotic development. If bacteria do not have resistance, they may develop or acquire resistance through contact with external genetic sources carrying resistance determinants, possibly from another species, by horizontal gene transfer (HGT) or through direct exposure to an antibiotic that induces selective pressure or vertical resistance [1,5,15]. Documented examples of HGT include the vancomycin-resistance-encoding gene *vanA* that originated from the *Enterococcus* species and was transferred to *Staphylococcus aureus* in Gram-positive bacteria. In Gram-negative bacteria, the carbapenemase-associated gene *bla*_OXA-48_ found in a hospital isolate of *Proteus mirabilis* was discovered to have been transmitted from *Klebsiella pneumoniae* from the same hospital [15,16]. The misuse of antibiotics in a medical context helps in the selection of resistant strains due to the exposure to sub-lethal doses of antibiotics. This can happen by not taking the antibiotic for the recommended time, selecting individuals with higher tolerance, or by using antibiotics too often and even when not necessary, exposing bacteria to the antibiotic without a clear therapeutic benefit [1]. The wastewater of pharmaceutical factories can contain high concentrations of antibiotics, further exposing environmental microorganisms to a selective pressure that induces resistance. Globalization has made it increasingly easy for those pathogens to spread across the world [17]. Also, some antibiotics like colistin have historically been used as feed growth promoters, making them ubiquitous. MDR bacteria have become so widespread to be present in wildlife that have minimal contact with humans [18].

Biofilms are an additional problem related to antibiotic resistance, as they significantly increase the risk associated with an infection and decrease susceptibility to antibiotics [14]. Biofilms are complex structures composed of microbial cells enclosed in hydrated extracellular and non-crystalline polymers, adhered to a surface, and used as a survival strategy [19,20]. It is estimated that over 80% of all human infections are associated with a biofilm [21]. The mutation rates and horizontal transmission of genes are more frequent in these structures, increasing the likelihood of the same bacteria to accumulate a resistance to several antibiotics. Depending on the biofilm structure, biofilms may limit the molecular diffusion to their inner-most cells, making only a small portion of the antibiotic rapidly available [5,22]. Further penetration is slow, reducing the maximum concentration at which cells are exposed and giving them time to produce resistance factors. A great deal is known about the resistance mechanisms that bacteria use in the planktonic state, but not so much is known about the resistance mechanisms in biofilms, further hindering their treatment [5,23].

Ultimately, antibiotic resistance is not a grand challenge for humanity, but rather a series of interconnected problems involving several areas that require action from microbiologists, ecologists, health care specialists, educationalists, policymakers, legislative bodies, agricultural and pharmaceutical industry workers, and the general public [24].

## 2. The Search for New (Natural) Antimicrobial Drugs—A Crisis

One of the most compelling things about the use of antibiotics is the relatively low price that enables them to be readily available to most people around the world. Newly discovered antibiotics, on top of having to compete with the ones that are already on the market like every other drug, are usually reserved for the cases when the existing antibiotics do not work, to slow down eventual bacterial resistance to the new molecules. Although such an approach makes sense from medical and public health points of view, it discourages the development of new drugs from for-profit organizations, as it substantially decreases their revenue in the limited time they have until the intellectual property becomes public domain. Zemdri (plazomicin), an aminoglycoside, was reported to have cost USD 750 M to enter the market in 2018 but made less than USD 800 k in sales in the first six months. Shortly after, the pharmaceutical company that developed it declared bankruptcy, and the intellectual property was sold for only USD 16 M [25]. In recent years, new financial models have started to emerge to incentivize the research and development of new antibiotics. However, despite the recognition of this problem, the implementation of incentives like government-funded market entry rewards has been gradual and restricted. After so much time has been neglected, the market is in dire need of new products [25]. Recent events including the COVID-19 pandemic and its aftermath as well as the war in Ukraine have introduced a new layer to the problem in Western countries, provoking shortages of staple first-line antibiotics, like amoxicillin, that are often produced as generic medicine in China. This single country has cornered the antibiotic market, which, given its importance, has become more problematic to Western policymakers that are now starting to try to take that industry back [26,27].

Natural products have always been a crucial part of the drug discovery pipeline. Tetracycline, an example of a natural antibiotic, was detected in ancient skeletal remains from the Roman period in Egypt. This particular antibiotic is easily preserved due to its strong chelator activity of the hydroxyapatite mineral portion of bones and tooth enamel. Other sources of antibiotics from the pre-antibiotic era have also been reported, such as those found in the red soils of Jordan and traditional Chinese medicine practices, but physical evidence is harder to collect [24]. Prontosil (sulfamidochrysoidine), Salvarsan (arsphenamine), and Neosalvarsan (arsphenamine methylenesulfoxylic acid sodium salt) were very successful antibiotic drugs from the beginning of the 20th century, but the antibiotic era truly started in the 1940s with the widespread availability of a penicillin medicine isolated from *Penicillium rubens* [24,28,29,30,31,32]. In the next decades, until the 1970s, in large part due to the development of a systematic screening approach, many more antibiotics were discovered, and most are still in use today, although the increased incidence of MDR bacteria has limited their use [33]. After this period, the “me-too” drugs became more frequent. These drugs take an existing drug and make a slight alteration that does not change its mode of action, but makes it less toxic or more effective [34]. Importantly, they only slightly delay the resistance of bacteria to a given drug class. For example, methicillin entered the market as a penicillin derivative invulnerable to degradation by penicillinases, which are one of the main pathogenic bacteria defense mechanisms. Unfortunately, less than two years after its introduction, the first methicillin-resistant *S. aureus* (MRSA) was reported, rapidly becoming the reference for hospital-acquired infections and spreading elsewhere [35,36]. We are currently living at the beginning of the post-antibiotic era, where antibiotics are much less effective than before [5].

**Figure 1:**
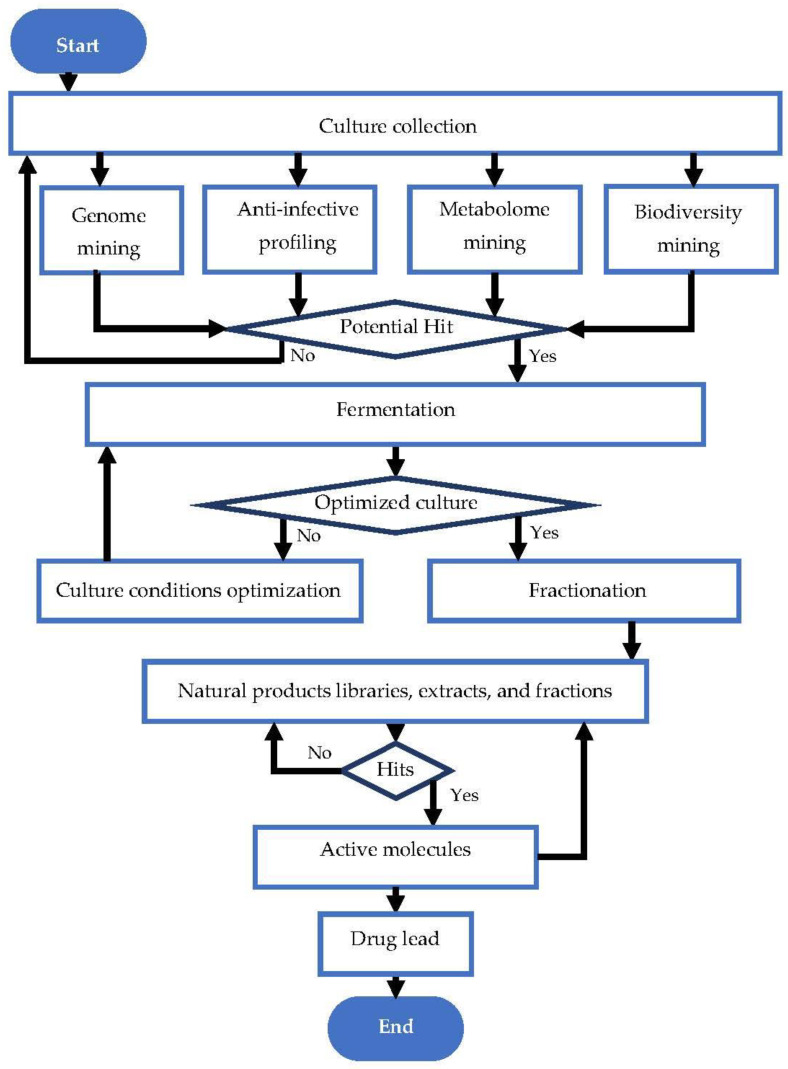
Flow chart of natural antibiotic discovery pipeline. Adapted from Karwehl and Stadler [37].

The discovery of natural antibiotic molecules is a well-established process that involves multiple steps, including the search for a promising bioactive molecule source, fermentation, optimization and up-scaling, fractionation, and identification of active molecules if the activity prevails. These steps are summarized in Figure 1.

Since the 1990s, natural drug discovery programs have slowed down due to a combination of technical limitations and legal hurdles. Technical limitations stem from the belief that natural drugs are incompatible with high-throughput screening approaches, which have become a cornerstone of modern drug discovery. Legal hurdles have emerged from the increased regulation in the industry due to the United Nations Convention on Biological Diversity, which aims to share the benefits arising from the fair and equitable utilization of genetic resources between the countries possessing them and the companies taking advantage of them [38,39,40]. These factors have made it increasingly challenging to develop new natural drugs, which has led to a decline in the number of natural products being used in drug discovery, instead favoring synthetic molecules. This strategy was not very successful at finding new drug leads in major therapeutic areas, including antibiotics. Almost all classes of commercial antibiotics used are natural or semi-synthetic; the exceptions are the sulfa drugs, quinolones, and oxazolidinones, and only the last of those is somewhat recent. Natural drugs are thus resurfacing, as they have a better track record [8,41]. They are more chemically diverse and often have a larger molecular weight than synthetic drugs. Unlike synthetic drugs, natural drugs are not discovered by testing the combinatorial-derived libraries of small molecules. They often do not follow Lipinski’s rule of five, which should affect absorption, distribution, metabolism, excretion, and toxicity (ADMET), despite being viable oral drugs [8,38]. The relative complexity and consequent specificity of certain biological drugs, like peptide therapeutics, even allow them to reach targets that were previously considered “undruggable” [41].

Secondary metabolites, which are molecules that increase the competitiveness of an organism without being essential to its survival, constitute the majority of relevant natural products. Historically, although penicillin is very famous for being a revolutionary discovery, most natural antibiotics are produced by actinobacteria [37]. Microbes play a growing role in the discovery of these products as they have provided effective drugs against viruses, bacteria, fungi, and even cancer [42].

The rediscovery of previously identified molecules poses a major bottleneck in natural product research, resulting in an increased workload that fails to yield meaningful results. Fortunately, there are newly available techniques like high-resolution mass spectrometry (HRMS) coupled with existing ones like nuclear magnetic resonance (NMR) spectroscopy and pre-fractionation methods like C_18_ high-performance liquid chromatography (HPLC) or liquid- and solid-state extractions. These techniques have allowed for the identification of replicated molecules at an early stage, thus mitigating this problem [39,43,44]. With such powerful tools becoming increasingly available for molecule identification, finding new sources of bioactive compounds becomes ever more fruitful.

## 3. Fungal Secondary Metabolites

The secondary metabolome of fungi is vast and largely unexplored, especially from Ascomycetes and Basidiomycetes, which comprise filamentous fungi [43]. They are the source of many compounds with medical, industrial, and agricultural importance [45].

**Figure 2:**
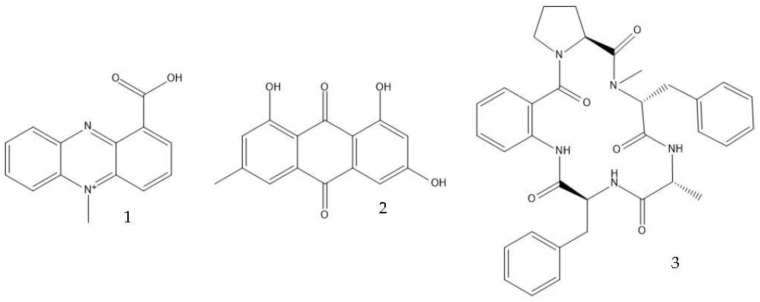
Structures of 5-methyl-phenazine-1-carboxylic acid (**1**), emodin (**2**), and avellanin C (**3**).

Filamentous fungi provide several ecosystem functions including the following: the symbiotic ones have a close, mutually beneficial relationship with a nonfungal organism; the saprobic ones utilize the nutrients released when breaking down debris; and parasitic fungi feed by taking away nutrients from another organism [46]. One strategy employed by several fungi to stay ecologically relevant is to produce antibiosis molecules, and some of them can be used as medicine [47]. The niches where they belong are very competitive, so on top of the pure weaponization of their secondary metabolome, they also produce a multitude of signal molecules that may have other valuable properties. For example, 5-methyl-phenazine-1-carboxylic acid (**1**) is an antifungal drug, but in the wild, it is used in smaller quantities as a signal to induce asexual sporulation [43,48]. Other fungal metabolites have different but potentially interesting effects, like emodin (**2**), which only has a moderate antibacterial effect but significantly enhances the efficacy of oxacillin against MRSA when combined with it [49], and avellanin C (**3**, Figure 2), which has anti-quorum sensing properties in *S. aureus* [50].

The three most important classes of secondary metabolites from fungi are polyketides, derived from acyl CoA precursors; non-ribosomal peptides that condense amino acids in pathways involving enzymes instead of ribosomes; and terpenoids formed by mevalonic-acid-derived units [51,52,53].

**Figure 3:**
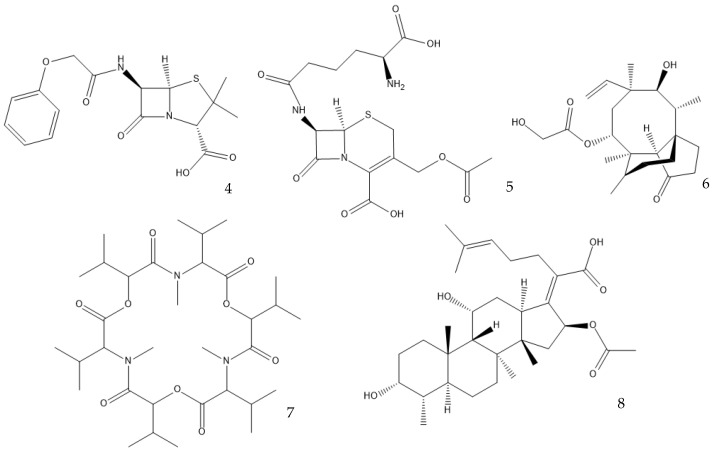
Structures of penicillin V (**4**), cephalosporine C (**5**), pleuromutilin (**6**), fusafungine (**7**), and fusidic acid (**8**).

Many antibiotics have been discovered from filamentous fungi. Penicillins like penicillin V (**4**, Figure 3) and cephalosporines like cephalosporine C (**5**) were the first and second classes that were discovered, respectively. They are chemically very similar, as both are non-ribosomal peptides that have a β-lactam ring (four-atom cyclic amide) that binds to peptidoglycans and prevents them from cross-linking, compromising the stability of the cell walls, especially from Gram-positive bacteria [1,46]. Pleuromutilin (**6**) and its derivatives have taken a long time to enter the human market despite being discovered in the “golden age” of antibiotics. They block the start of protein synthesis by interfering with the 50S bacterial ribosomal unit [8]. Enniatins like fusafungine (**7**) are produced by mycotoxin-producing *Fusarium* spp. They interfere with the cell wall and disrupt the cell membrane, but show other effects such as the inhibition of drug efflux pumps and MDR phenotype expression [33,54]. Fusidic acid (**8**) is the only member of its class and is a narrow-spectrum antibiotic that is commonly used against *S. aureus*. It binds to an elongation factor and the corresponding ribosome during protein synthesis, which does not allow it to continue protein synthesis. While eukaryotes have other elongation factors, bacteria become unable to synthesize proteins [55]. 

**Figure 4:**
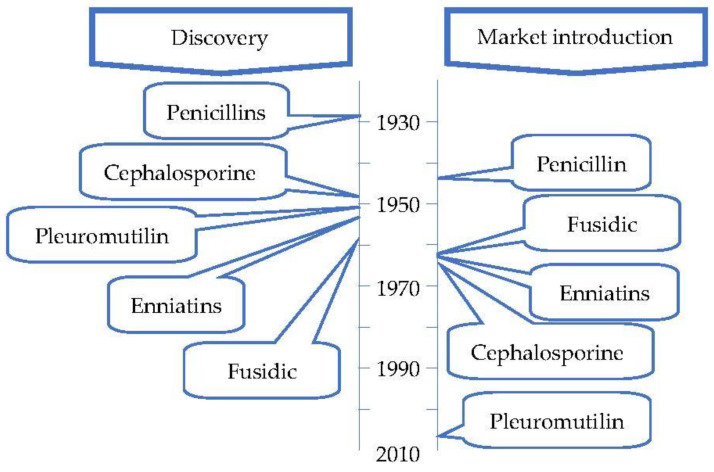
Timeline of the discovery and market entry of the first antibiotic of each class isolated from fungi (adapted from Hutchings et al. [33]).

Figure 4 contains a timeline of the discovery and market introduction of the antibiotic classes isolated from fungi.

One of the most challenging aspects of discovering new drugs from fungi is their production at a large scale, as standard laboratory conditions are often not suitable for that purpose [56].

## 4. Approaches to Secondary Metabolite Production from Fungi

### 4.1. OSMAC Approach

Culture conditions are of utmost importance in determining which compounds will be synthesized and in which quantities. There are countless examples of effects, but a relevant one is an extract of *Bulgaria inquinans*, which accumulated four new metabolites only when a mixture of inorganic salts was added to the culture [57]. Even the harmless change from tap water to distilled water in the making of the culture media promoted the production of six new secondary metabolites in a *Paraphaeosphaeria quadriseptata* culture [58]. The “One Strain MAny Compounds” (OSMAC) approach may be incredibly successful at enhancing the chemical diversity obtained, as changing culture conditions like the pH, temperature, aeration, and carbon and nitrogen sources can alter the metabolite profile of the fungi. Potato dextrose or malt extract are both great to maintain fungal cultures but often fail in terms of secondary metabolite production. Adding enzyme inducers/inhibitors or chemical elicitors can also change what is produced [56].

The discovery and commercialization of penicillin had a significant time gap, mostly due to the time it took to develop an effective large-scale production method. The first versions of this method used a broth consisting of lactose (3–4%), corn steep liquor (4%), CaCO_3_ (1%), KH_2_PO_4_ (0.4%), and antifoam (0.25%). Over time, penicillin was improved, but the main difference is the replacement of lactose by glucose or molasses at 10%, which are cheaper. Phenylacetic acid, or another molecule that may serve as a side chain, was also added, as its synthesis is the limiting step. Corn steep liquor is advantageous for its price and chemical diversity, containing large amounts of micronutrients, nitrogen, and side-chain precursors [59,60]. The maximum volume of the reactors used increased, as new technologies allow for better oxygen diffusion, which is essential for the production of most secondary metabolites, including penicillin, but oxygen diffusion is a challenge, as fungus significantly increases the viscosity of the medium [59]. The morphology acquired by the cells influences the production of secondary metabolites, and this aspect needs to be optimized. It was shown that the formation of pellets does not directly affect the ability of *Penicillium chrysogenum* to produce penicillin, but reduces the medium viscosity, which, in turn, increases the diffusion rate of respiratory gases, reducing energy costs and increasing productivity. The morphology can be influenced by diverse aspects, such as the spore density of the inoculum, the agitation speed, or the CO_2_ concentration [59,61,62]. The strain that is used also changed significantly, and all factors are important. This increased the concentration of penicillin obtained in the broth from 0.001 g/L in the year 1939 to the current concentrations of over 50 g/L [52,59].

### 4.2. Liquid- and Solid-State Fermentations

Secondary metabolites can be produced via fermentation in a liquid broth; via solid support, like the Lightweight Expanded Clay Aggregates (LECA) “nuts”; or most often, by the use of a gelling agent like agar media, which consistently gives good results [56].

Submerged fermentation is often preferred in industrial processes because it is easier to scale up, monitor, and automate. A large scale is often essential for industrial-scale viability, and contrary to solid-state fermentation, it avoids the build-up of temperature, pH, and O_2_ concentration gradients in large reactors [63]. The purification of the products also becomes easier, but it ends up generating a great quantity of wastewater [64]. Liquid fermentation is notably fast, usually lasting only 1–2 weeks, so it requires diverse equipment to manage in real time. Frequently, the constant influx of nutrients supplied by a fed-batch stream is required, as nutrients in a liquid medium are easy to access, and thus are rapidly consumed by microorganisms [59,64]. In mushroom fermentations, using a liquid-state system does not allow the formation of fruiting bodies that sometimes contain bioactive molecules [65].

Submerged fermentation at an industrial scale must carefully consider several physical, chemical, and biological parameters that can severely influence the success of the operation. Among them, agitation and aeration can significantly increase energy consumption. This energy adds to that released from the fungal metabolism, so it requires an effective cooling system to maintain an appropriate temperature for the microorganism [59,66,67]. At a laboratory scale, it is easier to maintain an adequate temperature and aeration, given that the volume of a reactor with its walls and bottom is much smaller than in an industrial fermenter. Additionally, a small pump can provide a relatively large volume of air. However, even these reactors may create concentration gradients and agitation dead zones [67]. These parameters must be carefully considered before scaling-up operations. It is common to express the agitation used in these experimental setups in rotations per minute (rpm), but this fails to precisely express the forces undergone by the broth. The use of the agitation Reynolds number or the mixing time is thus easier to compare between independent experiments, especially at different scales. It is important to consider that the fermentation broth of filamentous fungi is usually a viscous, non-Newtonian fluid, so its viscosity depends on the shear rate, and more heavily so when the mycelium does not grow in pellets [61,67,68]. In an *Aspergillus niger* culture, it was found that the optimal rotation frequency for pectinase production was 150 rpm. While a slower agitation did not promote a significant mixture, higher speeds increased the shear force experienced by the cells and decreased the enzyme activity, while still increasing the biomass production, up to 200 rpm [69]. Another study on the same species directed for the production of tannase demonstrated that the optimal agitation speed was 130 rpm for enzyme activity and 100 rpm for growth optimization [70].

**Figure 5:**
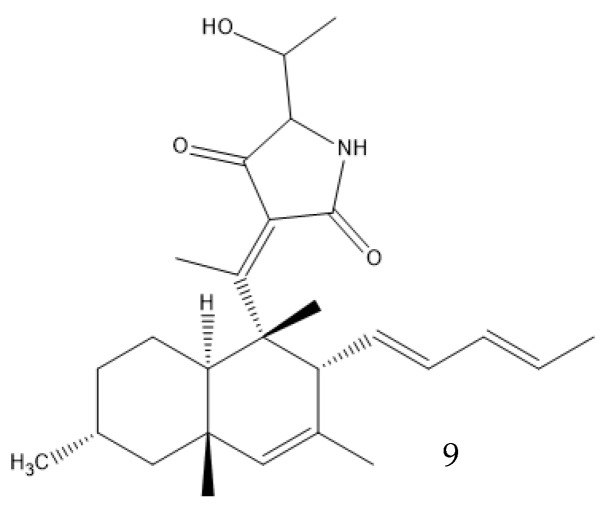
Coniosetin structure.

Solid-state fermentation may be described as occurring in a solid matrix that contains adequate moisture for the microorganism to develop, but no additional free water, which makes it optimal for organisms that thrive in low-water-activity environments. It can be performed in either an inert carrier or on insoluble substrates that supply the nutrients. It resembles many valuable fungi native environments, allowing organisms to better adapt to processual conditions. Both Basidiomycetes and Ascomycetes evolved on land, and only later did some species migrate to a marine environment. The marine microorganisms of these phyla thus often prefer solid media, as most of them are isolated from sediments or solid structures and do not freely swim in the water. It also removes the need for conditions that the fungi did not evolve to be adapted to, like high shear stresses and the presence of antifoam products. This type of culture promotes secondary metabolite production—for example, coniosetin (**9**, Figure 5), which is a potential antibiotic from *Coniochaeta ellipsoidea*, can only be produced in a solid state despite the mycelium also growing well in liquid media [63,71].

Solid-state fermentation is sometimes considered a low-technology approach, best suited to high-volume low-cost applications that require low purity like industrial enzymes—amylase, cellulase, protease, etc. However, this has been changing, and more valuable products have been produced this way, although bacteria and yeast are more commonly employed in such systems [72,73]. On a laboratory scale, it has many advantages such as higher productivity, concentration of products, and product stability (due to lower water activity), and lower catabolic repression and sterility requirements, but its use is hindered by the difficulty in scaling up [63].

### 4.3. Co-Culture Strategies

**Figure 6:**
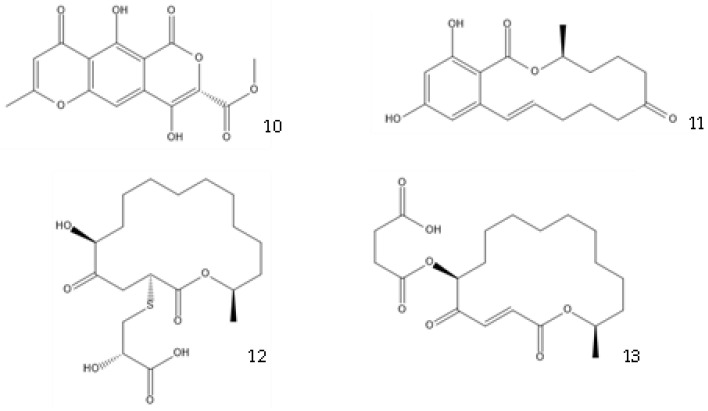
Structures of lateropyrone (**10**), zearalenone (**11**), berkeleylactone A (**12**), and A26771B (**13**).

Co-culture or mixed fermentation strategies may be of great value to unlock the expression of cryptic pathways, producing molecules that are absent in pure-strain cultures such as pheromones, defense molecules, or metabolites that are related to symbiotic associations. These strategies consist of growing different microorganisms in the same confined environment, allowing them to interact with each other [74]. Such interactions may even alter the core metabolism of the cells, for example, by increasing the production of ergosterol [75]. In addition to a yield increase in common molecules, these types of strategies can increase the yield of previously unidentified or undetected molecules, facilitating their characterization [76]. They can also help to overcome some problems that fermentative processes undergo, including excessive metabolic burden, a lack of active enzyme expression, and by-product formation [77]. This makes sense from an ecological perspective, as the production of antibiosis molecules by a fungus is most important when exposed to a competitor [37]. A culture of *Fusarium tricinctum* and *Streptomyces lividans* produced lateropyrone (**10**, Figure 6) and zearalenone (**11**), which are two antibacterial molecules, only when co-cultivated [78]. Berkeleylactone A (**12**) and A26771B (**13**), two macrolide antibiotics, were also obtained only when two extremophilic *Penicillium* species—*P. fuscum* and *P. camembertii/clavigerum*—were co-cultivated [79].

To co-cultivate bacteria and fungi for drug discovery, the most common strategy is similar to regular monoculture fungi fermentations, but the liquid medium is inoculated with a small number of bacteria, often not at the same time as the fungi. Similarly, solid media like commercial rice or wheat can also be used. The bacteria should not dominate the culture but, at a small scale, can be regulated by the inoculum ratio [74,80,81]. An alternative proposed method grows the fungi in glass beads submerged in a culture medium. After the fungus has developed, the medium is replaced with a bacterial suspension, but the mycelium remains adhered to the beads, establishing a mixed culture [82]. These methods, however, have some limitations. Firstly, they are not employed more often because they require laboratory expertise in the cultivation of both fungi and bacteria [74]. Additionally, the induced metabolites that are produced are quite random. For example, when the antimicrobial activity of a culture of *Streptomyces* sp. (which, despite being a bacterium, has many similarities with fungi in terms of antibiotic production) was tested against human pathogens, no significant effect was found against *P. aeruginosa*. A co-culture with *P. aeruginosa* increased the 4-fold activity against all the Gram-positive bacteria tested, but, unexpectedly, did not increase the activity against that pathogen [83]. In the production steps of the drug development pipeline, especially while scaling up the reactor, it can also be difficult to keep the co-culture stable. In this regard, some methods can help to surpass this limitation, like a pulsating feed of glucose that gives a fitness advantage to alternating species. [84].

### 4.4. Other Optimization Strategies

**Figure 7:**
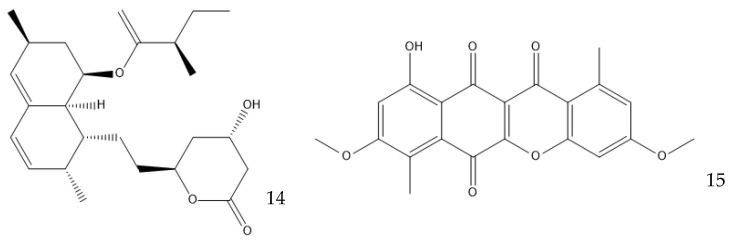
Structure of lovastatin (**14**) and bikaverin (**15**).

Many other conditions can be varied to optimize the production of a metabolite, which are often limited by the knowledge of the mechanisms involved. Surfactants can have an interesting effect on liquid fermentation. It was found that the yield of lovastatin (**14**, Figure 7) production by *Monascus purpureus* was increased by 84.5% by the addition of Triton X-100, a nonionic surfactant. This surfactant increases the membrane permeability, facilitating the export of the product of interest and increasing the expression of the genes related to lovastatin synthesis [85,86]. The addition of Tween 80 on a *Ganoderma lucidum* culture, which produces bioactive exopolysaccharides (EPS), or on a *Coprinopsis cinerea* culture, which produces cellulose, also increased their yield [87,88].

The inclusion of polymers like polyacrylic acid or sodium polyacrylate modifies the spores’ surfaces to avoid the formation of clumps and makes the mycelia grow in dispersed hyphal filaments [89]. Another exhaustive study used either XAD-16N or styrene-divinylbenzene, two resins, to induce the production of many compounds that were not present otherwise in fermentation with 349 different fungal strains [90]. Adsorptive polymeric resins can increase biomass growth and drastically change the color of the fermentation broth. They interfere with the fermentation by sequestrating components from the medium. Those might be microelements or toxic products, which are especially relevant if the product of interest is extracellular and toxic or degrades easily [91,92].

Some small molecules may serve as epigenetic modifiers by interfering with DNA methyltransferase, like 5-azacytidine and 5-aza-20-deoxycytidine, or with histone deacetylase, like compounds with hydroxamic acid or cyclic peptides (e.g., trichostatin A and trapoxin B) [91]. This may induce the expression of metabolic pathways that are cryptic in standard laboratory conditions, as those enzymes are responsible for the regulation of gene expression. Although the production of secondary metabolites is enhanced, this method is usually not specific to certain secondary metabolites. However, it was found that hydrazine boosted the production of some specific metabolites in a *Dothiora* sp. culture, including the antibiotic fusidic acid (**8**), and inhibited others [93]. Similarly, while reducing histone deacetylase activity using a mixture of compounds, *Aspergillus nidulans* overexpressed and under-expressed similar numbers of secondary metabolites by at least two orders of magnitude [94].

Using microparticles of talc (e.g., magnesium silicate), aluminum oxide, titanium oxide, or silver nanoparticles is another strategy to enhance the production of fungal products, specifically enzymes [95]. Talc microparticles have been shown to increase lovastatin production by *Aspergillus terreus* by 60%, although this effect is due to their ability to morphologically engineer the fungus. By reducing the pellet diameter, they increased the oxygen availability in the inner part of the pellets and, consequently, the product yield [96,97]. Aluminum oxide microparticles increased the amylase production by 114% in an *Aspergillus oryzae* fermentation through a similar mechanism [95].

There are even other parameters that can be changed to improve the production of antibiotics. Starving the cells of a nutrient can be effective in specific cases; for example, in the production of the polyketide bikaverin (**15**) by *Fusarium oxysporum*, nitrogen, phosphorous, and sulfate limitations increase the yield, while the industrial production of penicillin by *P. chrysogenum* must have phosphates as the limiting nutrient [59,98]. Secondary metabolites are produced in the stationary phase of cellular growth, which usually happens when there is a sugar limitation [99,100]. The pH can play a part in the production of secondary metabolites by regulating the expression of enzymes that are involved in their production. *A. nidulans* favors the production of penicillin in alkaline environments because the gene encoding the isopenicillin N synthetase, which is responsible for the production of an intermediate in the penicillin biosynthetic pathway, is up-regulated in these conditions [101]. This effect is observed in *P. chrysogenum*, but to a lesser extent [100]. Bikaverin production, on the other hand, is favored by an acidic pH [98].

Table 1 shows examples of fungal metabolites with antibacterial properties, their structure, the producing species, and the culture media used.

The optimization of culture conditions can have an improved hit rate when using the ever-increasing available information about the biosynthetic pathways involved in the production of metabolites [117]. While some parameters should be optimized based on the producing species, others are specific to a particular pathway and must be established accordingly.

## 5. Biosynthetic Pathways for Antibiotic Production

Computational tools have been of great utility as a first step in the search for new secondary metabolic pathways, especially with the developments of large-scale sequencing technologies [117]. One such tool is genome mining for biosynthetic gene clusters (BGCs), which enables the identification of strains which contain genes encoding for the enzymatic machinery that is required to produce bioactive compounds. Unlike the sequences related to primary metabolites, which are spread through the genome, the ones related to the same secondary metabolite usually appear together, in a cluster, and can be identified using appropriate algorithms [43]. The enzymes encoded within these clusters can be used to predict the metabolites that they produce, based on known analogies [118]. However, traditional laboratory techniques are still required to properly identify the active molecule [119].

BGCs are very common among fungal species. For instance, in one study performed by de Vries et al. [120], the genomes of 19 *Aspergillus* species were analyzed, and it was verified that they contained between 21 and 66 BGCs each. Considering the millions of fungal species estimated to exist, many of which with a number of BGCs in that range, the potential for biosynthetic pathways is enormous [121,122].

The Kyoto Encyclopedia of Genes and Genomes (KEGG) provides a brief look at the biosynthetic potential of a microorganism. However, it lacks information about many less-common metabolites and fungal species. It is still a great and easy-to-use tool, but only when it is known which metabolite production is intended. If any fungal species is to be checked for penicillin or cephalosporin production, the consultation of “Biosynthesis of secondary metabolites” from *P. rubens* quickly points to the probable production of penicillin from L-cysteine, L-valine, and lysine. However, the combination of only two restrictions (antibiotics from a particular species, for example) is often sufficient to prevent any results from being produced. This means that many compounds are not even present in the pathway. Many pathways are also incomplete for several reasons [123], even when the microorganism is widely reported to produce the end metabolite, like the penicillin synthesis by *P. rubens* [124]. Other pathways have an isolated hit because of an enzyme that is used for multiple substrates. Even if the pathway is accurate and complete, using its information to boost the production of a metabolite may be misleading. Taking penicillin again as an example, the use of the abovementioned amino acids to boost production will probably be unfruitful, as the limiting step in its synthesis is related to the side chain, and not the penicillin scaffold [125]. The availability of L-cysteine is only an important factor for achieving high yields of penicillin production in industrial strains that have already been optimized for side-chain synthesis. For those cases, having an adequate supply of L-cysteine is crucial for maximizing the efficiency of the production process [52]. This kind of metabolic engineering is required to obtain a desired product that is economically viable.

## 6. Advances in Metabolic and Genetic Engineering of Filamentous Fungi

The disparity between the number of BGCs identified using bioinformatic tools and the actual number of secondary metabolites produced suggests that many of these clusters remain silent under the conditions that are typically used in laboratory settings. Other molecules may even be produced, but at such low titers that they cannot be analytically detected, and their bioactivity is masked by more abundantly produced metabolites [42,57]. The metabolic engineering of fungi is one of the most important research directions in modern industrial biotechnology.

**Figure 8:**
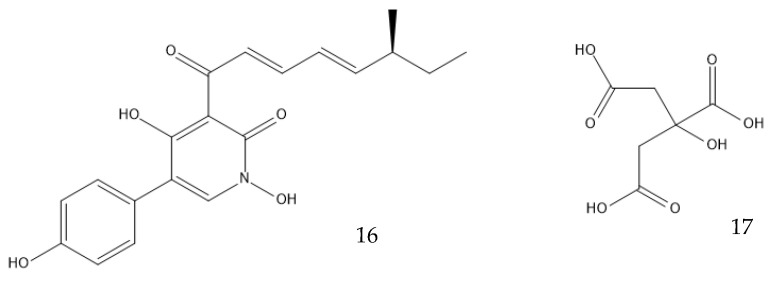
Structures of tenellin (**16**) and citric acid (**17**).

Genetically modifying filamentous fungi is possible, but much harder than genetically modifying bacteria or yeasts [52]. *Escherichia coli*, *Saccharomyces cerevisiae*, and *Komagataella phaffii* have successfully been turned into efficient cell factories, but the equivalent does not exist for molds nor mushrooms. Common industrial “workhorse” species include *A. niger*, *A. oryzae*, *P. chrysogenum*, *Trichoderma reesei*, and more recently, *Myceliophthora thermophila.* They use cheap feedstock, have powerful secretory pathways, and can post-translationally modify proteins. They are, however, still not as easy to engineer as bacteria or yeast because of their slow growth rate, low throughput of genetic transformation, and secretion of unwanted enzymes [126]. Bioprospecting new strains with optimized properties in which valuable BGCs can be inserted may be facilitated with the use of the fast-expanding genomics data, for example, from the Joint Genome Institute’s (JGI) Mycocosm database. Even if imperfect, the ones that are currently available can produce great results; the heterologous production of tenellin (**16**) in *A. oryzae* has a five-fold increased yield when compared with the native host of the biosynthetic pathway, *Beauveria bassiana* [126,127,128]. Alternatively, strain improvement through breeding tools that select desired traits or genetic engineering may provide a suitable strain [52,126]. An *A. niger* strain that produced 31 g/L of citric acid (**17**, Figure 8) underwent UV-induced mutagenesis, and the yield increased to 50 g/L. Further improvement using n-methyl-n-nitro-n-nitroso guanidine (MNNG), which is a chemical mutagenic, increased the yield to 96 g/L, and substrate optimization made it reach 114 g/L [129]. A different strain of the same species was genetically engineered to overexpress a high-affinity glucose transporter (HGT1). It boosted the production of citric acid from 162 g/L to 174 g/L by increasing the glucose usage [130]. Genomics, transcriptomics, proteomics, metabolomics, and localizomics, in addition to the proper identification of the biochemical pathways, are the keys to inducing or inhibiting the expression of a secondary metabolite [52]. New algorithms using machine learning and artificial intelligence may help to combine all the omics data obtained from the ever-increasing throughput methods. They might discover ways to produce new industrially viable biological products via the activation of silent BCGs by referring to an extensive knowledge of the genomes of many species, or they might discover better ways to produce those that already exist, for example, by avoiding the formation of an inconvenient secondary product. The data required for all of this may even be already discovered. Having a comprehensive understanding of all the mentioned fields is essential to develop a new generation of filamentous fungal chassis that can serve as chemical cell factories in synthetic biology. Such a platform would offer precise control over regulation at all levels, providing significant potential for enhancement [52,131,132].

Industrially viable penicillin-producing strains contain many examples of the application of such techniques, as the associated product has been under constant improvement for many decades [59]. In penicillin production, the excess presence of the precursor phenylacetic acid up-regulates the expression of genes in the homogentisate pathway that are involved in its degradation. Although that makes sense for the fungi, as it can reduce the concentration of this very toxic metabolite, it diminishes the amount of compound that is available to be incorporated into the penicillin molecule. Industrial strains thus have this catabolic pathway inactivated to avoid the waste of a chemical that constitutes one of the major costs of the process and to overproduce penicillin. Those strains have more copies of penicillin-associated BGCs; a higher transcription of these genes and the genes that are associated with the precursor amino acid’s production (L-cysteine, L-valine, and lysine); the inactivation of pathways that compete with their synthesis, such as the tryptophane synthesis; an increased number of peroxisomes, where one of the reactions take place; as well as the inactivation of the synthesis of a pigment that is considered a contaminant in downstream processing, among other mutations [59,133,134]. The mutations in industrial penicillin-producing strains are the accumulation of many years of improvement using several methods, and many lessons can be learned from them. They can be used as an inspiration for strain improvement in other processes using modern technology [52].

## 7. Conclusions and Future Directions

Antibiotic resistances and biofilm-related infections are among the most challenging problems of the next few decades, and if current trends continue, infectious diseases may once again become common death causes. As such, the combined action of governmental and civil society entities is required to implement policies that incentivize drug discovery for the sustainable management of this growing problem. Fungi have the potential to play a major role in this regard, given their diverse secondary metabolome, which is now more accessible with the recent advancements in genomics and metabolomics as well as new cultivation techniques. Natural products have become easier to screen using analytical techniques like mass spectrometry, regaining relevance in drug discovery, and fungi are historically valuable sources of such products. The technology involved in the production of valuable fungal products has also been improving rapidly, increasing their feasibility. The future of this line of research looks even more promising as new fungal species are constantly being identified and may produce valuable compounds. As computational power exponentially increases and allows for the development of more accurate in silico models, possibly involving artificial intelligence and using very large amounts of different data, there will be increasingly accurate predictions on where to look for and how to produce valuable metabolites. Developing a model filamentous fungus that is very well studied and highly regulable is also a priority to allow for the expression of BGCs that originate from more fastidious organisms.

## Figures and Tables

**Table 1:** Examples of fungal metabolites with antibacterial properties. Unless otherwise stated, the collected studies were relatively small-scale, conducted in liquid media, and used monoculture fermentation.

Antibiotic	Producing Species	Structure	Media Used	Observations	References
Penicillin G	*Penicillium chrysogenum*	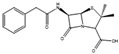	Glucose or molasses, 100 g/L; corn steep liquor solids, 45 g/L; phenylacetic acid, 0.65 g/L (fed continuously); vegetable oil–antifoam, 0.5 g/L; ammonium sulfate (continuously kept at 275 g/L).	Media for industrial production	[59]
Bis-N-norgliovictin	*Asteromyces cruciatus*	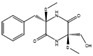	Glucose, 30 g/L; arginine, 1 g/L; asparagine, 2.5 g/L; glutamate, 1.5 g/L; FeSO_4_, 0.01 g/L; KCl, 0.5 g/L; MgSO_4_, 0.5 g/L; K_2_HPO_4_, 1.0 g/L; in artificial sea water.		[102]
Copsin	*Coprinopsis cinerea*	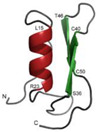	Glucose, 5 g/L; asparagine, 2 g/L; adenine sulfate, 50 mg/L; KH_2_PO_4_, 1 g/L; Na_2_HPO_4_, 2.3 g/L; Na_2_SO_4_, 0.3 g/L; ammonium tartrate, 0.5 g/L; thiamine-HCl, 40 μg/L; MgSO_4_·7H_2_O, 0.25 g/L; p-aminobenzoic acid, 5 mg/L.	Cultured in glass beads; co-cultured with *Bacillus subtilis* or *Escherichia coli*	[82,103]
Trichogin GA IV	*Trichoderma longibrachiatum*	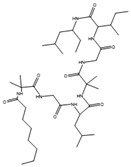	Glucose, 5 g/L; potassium dihydrogen phosphate, 0.8 g/L; potassium nitrate, 0.72 g/L; calcium phosphate, 0.2 g/L; magnesium sulfate, 0.5 g/L; manganese sulfate, 0.01 g/L; zinc sulfate, 0.01 g/L; copper sulfate, 0.005 g/L; iron sulfate, 0.001 g/L.		[104,105]
Averufanin	*Aspergillus carneus*	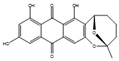	Glucose, 10 g/L; mannitol, 20 g/L; sucrose, 20 g/L; yeast extract, 3 g/L; corn syrup, 1 g/L; peptone, 10 g/L; tryptophan, 0.5 g/L; K_2_HPO_4_, 0.5 g/L; MgSO_4_·7H_2_O, 0.5 g/L; FeSO_4_·7H_2_O, 0.1 g/L; agar, 15 g/L.	Extracted from the solid culture medium	[106]
Oxasetin	*Vaginatispora aquatica*	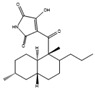	Extract from potato, 4 g/L; glucose, 20 g/L. pH adjusted to 7.		[107]
Bovistol D	*Coprinopsis strossmayeri*	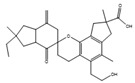	Extract from potato, 4 g/L; glucose, 20 g/L.		[108]
Illudin I	*Coprinopsis episcopalis*	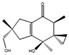	Sucrose, 80 g/L, yellow corn meal, 50 g/L; yeast extract, 1 g/L.		[109]
Aspergicin	Two *Aspergillus* spp.	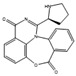	Glucose, 10 g/L; yeast extract, 1 g/L; peptone, 2 g/L; crude sea salt, 3.5 g/L.	Yielded another antimicrobial compound	[76]
Emericellin A	*Emericella* sp.	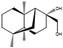	Peptone, 1 g/L; malt extract, 20 g/L; sucrose, 20 g/L.	Another similar compound was also isolated	[110]
Palmarumycin C_8_	*Lophiotrema* sp.	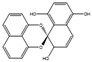	Glucose, 20 g/L; maltose, 10 g/L; yeast extract, 4 g/L; oatmeal, 20 g/L; 5-azacytidine, 12 mg/L.		[111]
Diaporthin	*Diaporthe terebinthifolii*	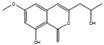	Malt extract, 20 g/L; glucose, 20 g/L; peptone, 1 g/L.	Other media also yielded this and another antimicrobial compound	[112]
Phomopsin A	*Phomopsis* sp. ZSU-H76	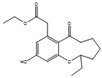	Glucose, 10 g/L; peptone, 2 g/L; yeast extract, 1 g/L; NaCl, 3 g/L.	Other compounds were also isolated	[113]
Eugenol	*Neopestalotiopsis* sp. MFLUCC15-1130	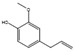	Extract from potato, 4 g/L; glucose, 20 g/L.		[114,115]
3-phenylpropionic acid	*Cladosporium cladosporioides*	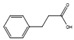	Extract from potato, 4 g/L; glucose, 20 g/L.	Other compounds with weaker antimicrobial effects were also identified	[116]

## Data Availability

Data sharing not applicable.
