# Correction: Correia et al. Beyond Penicillin: The Potential of Filamentous Fungi for Drug Discovery in the Age of Antibiotic Resistance. 2023, *12*, 1250

**DOI:** 10.3390/antibiotics13100944

**Published:** 2024-10-09

**Authors:** João Correia, Anabela Borges, Manuel Simões, Lúcia C. Simões

**Affiliations:** 1 LEPABE—Laboratory for Process Engineering, Environment, Biotechnology and Energy, Faculty of Engineering, Department of Chemical Engineering, University of Porto, 4200-465 Porto, Portugal; up201806894@edu.fe.up.pt (J.C.); apborges@fe.up.pt (A.B.); mvs@fe.up.pt (M.S.); 2 ALiCE—Associate Laboratory in Chemical Engineering, Faculty of Engineering, University of Porto, 4200-465 Porto, Portugal; 3 CEB—Centre of Biological Engineering, University of Minho, Campus de Gualtar, 4710-057 Braga, Portugal; 4 LABBELS—Associate Laboratory in Biotechnology, Bioengineering and Microelectromechanical Systems, 4710-057 Braga, Portugal

Manuel Simões was included as a corresponding author in the original publication [1] by mistake. The corrected corresponding author should be Lúcia C. Simões, and the email is updated accordingly. The authors state that the scientific conclusions are unaffected. This correction was approved by the Academic Editor. The original publication has also been updated.
